# Changes in the Composition and Function of Lipoproteins after Bariatric Surgery in Patients with Severe Obesity

**DOI:** 10.3390/jcm10081716

**Published:** 2021-04-16

**Authors:** Idoia Genua, Núria Puig, Inka Miñambres, Sonia Benítez, Pedro Gil, Margarida Grau-Agramunt, Andrea Rivas-Urbina, Carme Balagué, Sonia Fernández-Alanin, Álvaro García-Osuna, Antonio Pérez, José Luis Sánchez-Quesada

**Affiliations:** 1 Endocrinology and Nutrition Department, Hospital de la Santa Creu i Sant Pau, 08041 Barcelona, Spain; igenua@santpau.cat (I.G.); iminambres@santpau.cat (I.M.); pgimll@icloud.com (P.G.); 2 Bellaterra Campus, Universitat Autònoma de Barcelona, Cerdanyola del Vallès, 08041 Barcelona, Spain; npuigg@santpau.cat (N.P.); arivas@santpau.cat (A.R.-U.); cbalague@santpau.cat (C.B.); 3 Cardiovascular Biochemistry Group, Research Institute of the Hospital de la Santa Creu i Sant Pau (IIB Sant Pau), 08041 Barcelona, Spain; sbenitez@santpau.cat (S.B.); mgrauag@santpau.cat (M.G.-A.); agarciao@santpau.cat (Á.G.-O.); 4 CIBER of Diabetes and Metabolic Diseases (CIBERDEM), Instituto de Salud Carlos III (ISCIII), 28029 Madrid, Spain; 5 General and Digestive Surgery Department, Metabolic & Bariatric Surgery Unit, Hospital de la Santa Creu i Sant Pau, 08041 Barcelona, Spain; sfernandeza@santpau.cat

**Keywords:** obesity, bariatric surgery, lipoproteins, lipoprotein function, LDL, HDL, apolipoproteins

## Abstract

The effect of bariatric surgery on lipid profile and the qualitative characteristics of lipoproteins was analyzed in morbidly obese subjects. Thirteen obese patients underwent bariatric surgery. Plasma samples were obtained before surgery and at 6 and 12 months after the intervention. Thirteen healthy subjects comprised the control group. Lipid profile, hsCRP, and the composition and functional characteristics of VLDL, LDL, and HDL were assessed. At baseline, plasma from subjects with obesity had more triglycerides, VLDLc, and hsCRP, and less HDLc than the control group. These levels progressively normalized after surgery, although triglyceride and hsCRP levels remained higher than those in the controls. The main differences in lipoprotein composition between the obese subjects and the controls were increased apoE in VLDL, and decreased cholesterol and apoJ and increased apoC-III content in HDL. The pro-/anti-atherogenic properties of LDL and HDL were altered in the subjects with obesity at baseline compared with the controls, presenting smaller LDL particles that are more susceptible to modification and smaller HDL particles with decreased antioxidant capacity. Bariatric surgery normalized the composition of lipoproteins and improved the qualitative characteristics of LDL and HDL. In summary, patients with obesity present multiple alterations in the qualitative properties of lipoproteins compared with healthy subjects. Bariatric surgery reverted most of these alterations.

## 1. Introduction

Obesity is a major public health issue that has become increasingly prevalent worldwide [1]. Patients with obesity have an increased cardiovascular (CV) risk [2], mostly because of the presence of comorbidities, such as diabetes, hypertension, or dyslipidemia. The chronic inflammation and increased oxidative stress present in obesity may also contribute to the increased CV risk [3]. One of these obesity-related comorbidities is the alteration in lipid profile. Atherogenic dyslipidemia in patients with obesity is characterized by an increased serum concentration of triglycerides and decreased high-density lipoprotein cholesterol (HDLc) levels [4]. In addition to the quantitative alterations found in obese subjects, these patients have alterations in the characteristics of lipoproteins, displaying small LDL and HDL particles, and large VLDL particles [5]. These qualitative changes in lipoproteins may alter their functionality, contributing to the increased CV risk [6].

Bariatric surgery lowers CV mortality [7], and the improvement of atherogenic dyslipidemia has been proposed to explain the benefits on CV outcomes after bariatric surgery [8]. The improvements in lipid profile after surgery include a decrease in triglycerides and an increase in HDLc [8,9], as well as qualitative and functional modifications to lipoprotein particles [10]. The potential benefits on lipoprotein composition, size, and functionality after bariatric surgery might contribute to lowering the risk of CV disease, and, consequently, the focus has shifted from studying the levels of lipoproteins to studying the quality of lipoproteins.

However, information about the effect of weight loss on the composition and functionality of lipoproteins is limited. Different studies have reported the alteration of different properties of both HDL and LDL. Most of these studies focused on the cholesterol efflux capacity of HDL, and an improvement in cholesterol efflux after bariatric surgery was reported by most [11,12,13,14], but not all [15], authors. The distribution of HDL subspecies has also been extensively studied in obese patients, and there is a consensus regarding the shift of HDL subfractions from small to large HDL particles after bariatric surgery [10,16]. However, other properties of HDL in obese subjects, such as its antioxidant capacity, have only been analyzed in one study [15], and there is scarce information regarding changes in HDL composition after bariatric surgery.

Regarding LDL, few studies have analyzed the qualitative properties of this lipoprotein, and they focused mainly on LDL size and plasma levels of oxidized LDL (oxLDL). There are contradictory reports on the effect of bariatric surgery on LDL size, with some studies indicating increased size after surgery [17,18], and others reporting no change [16,19]. Some studies described an increased concentration of oxLDL in obesity, which decreases after bariatric surgery [16,19,20,21]. However, other characteristics of LDL, such as lipid composition, the content of minor apolipoproteins, susceptibility to oxidation or aggregation, and the proportion of modified LDL particles in blood, have not been studied in obese subjects after bariatric surgery. The scarcity of studies on LDL is even more evident in the case of VLDL, beyond its known larger size in obese individuals with dyslipidemia [22].

Most previous studies focused on a specific lipoprotein fraction, so determining how alterations in one lipoprotein can affect other lipoproteins has not been possible. This aspect is relevant, as the metabolism of the different lipoproteins is closely interconnected, determining the characteristics and catabolism of VLDL and the properties of LDL and HDL. Furthermore, there is a close interaction between LDL and HDL in blood, and the exchange of lipids and apolipoproteins between LDL and HDL generates particles with greater or lesser atherogenic potential [23,24]. For this reason, carrying out a complete analysis of all lipoprotein fractions is necessary to uncover the mechanisms by which bariatric surgery modulates the pro- or anti-atherogenic properties of lipoproteins. The aim of this study was to evaluate the lipid profile, conduct an exhaustive analysis of the composition and function of the different lipoprotein fractions in patients with morbid obesity before and after bariatric surgery, and compare the data with those of a normal-weight healthy control group.

## 2. Materials and Methods

### 2.1. Study Population

Thirteen morbidly obese patients were included in the study. The inclusion criteria were patients with severe morbid obesity who underwent bariatric surgery at Hospital de Sant Pau. All patients met the criteria used in usual clinical practice for bariatric surgery in our clinic: body mass index (BMI) > 40 Kg/m^2^ or BMI > 35 Kg/m^2^ with comorbidities, including hypertension, diabetes, and hyperlipidemia. All subjects were free of inflammatory and infectious diseases, and none of them were receiving anti-obesity or anti-inflammatory drugs. The exclusion criteria were undergoing treatment with anti-inflammatory drugs or a prior diagnosis of a disease affecting lipid metabolism. Obese patients underwent bariatric surgery (sleeve gastrectomy or gastric bypass according to routine clinical practice), and blood samples were obtained one week before the intervention (baseline) and at 6 and 12 months after. Blood was collected in EDTA-containing Vacutainer tubes after an overnight fast of 12 h. Plasma was obtained by centrifugation at 1500 g for 15 min at 4 °C, and samples were frozen at −80 °C until analysis.

Thirteen healthy subjects were included in the control group. The controls were of normal weight, normolipidemic, and normoglycemic, and had no antecedents of inflammatory, infectious, or coronary disease or other diseases known to affect lipid metabolism. The clinical and anthropometric data of all groups are shown in Table 1. All subjects gave written informed consent before participating in the study, and the protocol was approved by the ethical committee of the Hospital de la Santa Creu i Sant Pau. The study was performed in accordance with the Helsinki Declaration.

### 2.2. Plasma Determinations

Lipid profile, C-reactive protein (CRP), total apoJ, Lp-PLA_2_ activity, LDL size, and HDL subfraction proportion were determined in plasma obtained in EDTA-containing Vacutainer tubes. The lipid profile included total cholesterol (Roche), triglycerides (Roche), apoB (Roche), and VLDL, LDL, and HDL cholesterol. Cholesterol of lipoprotein fractions was quantified using a direct HDL-cholesterol method (HDL-C plus, Roche) or by ultracentrifugation when the TG concentration was higher than 3 mmol/L, according to the National Cholesterol Education Program [25]. Non-esterified fatty acids (NEFA) were quantified using a commercial kit (Wako Pure Chemical, Osaka, Japan). All these determinations and CRP (Roche Diagnostics, Basel, Switzerland) were performed in a Cobas 6000/c501 autoanalyzer. ApoJ plasma levels were measured with commercial ELISA kits (Mabtech, Stockholm, Sweden). LDL size and HDL subfraction proportions were evaluated from plasma by non-denaturing polyacrylamide gradient (2.5%–16%) gel electrophoresis, as described previously [26]. Lp-PLA_2_ activity was determined using 2-thio-PAF (Cayman Chemicals, Ann Arbor, MI, USA) as a substrate [27] according to the manufacturer’s instructions. The distribution of Lp-PLA_2_ between lipoprotein fractions was measured by precipitating apoB-containing lipoproteins from plasma with dextran sulfate [28].

### 2.3. Lipoprotein Isolation and Composition

Lipoproteins were isolated from thawed plasma by flotation sequential ultracentrifugation, according to density: VLDL (1.006–1.019 g/mL), LDL (1.019–1.063 g/mL), and HDL (1.063–1.210 g/mL). Their lipid and apolipoprotein composition was determined by measuring the content of cholesterol, triglycerides, apoB, apoA-I (Roche Diagnostics, Basel, Switzerland), phospholipids, free cholesterol (Wako Pure Chemical, Osaka, Japan), apoA-II, apoE, and apoC-III (Kamiya Biomedicals, Seattle, WA, USA) in the autoanalyzer. ApoJ was evaluated using commercial ELISA (Mabtech).

### 2.4. Lipoprotein Functional Assays

Electronegative LDL (LDL(-)). The proportion of LDL(-) was quantified from the total LDL using stepwise anion exchange chromatography in a MonoQ 5/50 GL column (GE Healthcare, Chicago, IL, USA), as described previously [29].

LDL susceptibility to aggregation. LDL was dialyzed against phosphate-buffered saline (PBS) pH 7.4 using gel filtration chromatography in a PD10 column (Sephadex G-25, GE Healthcare). Aggregation of LDL was induced by SMase lipolysis. LDL (0.6 mmol/L cholesterol) was incubated at 37 °C for 1 h with SMase from *Bacillus cereus* sp. (Sigma Diagnostics, Livonia, MI, USA) with a final concentration of 50 mU/mL in the presence of 2 mM CaCl_2_ and 2 mM MgCl_2_. Size-exclusion chromatography was performed for monitoring aggregation using a Superose 6 Increase 5/150 GL column in an AKTA-FPLC system (GE Healthcare), as described [30].

LDL and HDL susceptibility to oxidation. LDL and HDL were dialyzed against PBS pH 7.4 using gel filtration chromatography in a PD10 column (Sephadex G-25, GE Healthcare). Susceptibility to oxidation was evaluated by monitoring the formation of conjugated diene at 234 nm in a Synergic HT spectrophotometer (BioTek). LDL or HDL at 0.15 mM of cholesterol was incubated with 5 µM of CuSO_4_, and the lag phase time of the oxidation kinetics was determined, as described [31].

Antioxidant capacity of HDL. HDL at 0.15 mM of cholesterol was incubated with a standard LDL (obtained from a pool of normolipidemic plasma and stored with 10% sucrose at −80 °C), and oxidation was induced by adding 5 µM of CuSO_4_. Conjugated diene formation was monitored, as explained in the LDL section. The results were expressed as the capacity of HDL to prolong the lag phase time of the standard LDL alone, as described previously [32]. See Supplementary Materials for details.

### 2.5. Statistical Analysis

Statistical analysis was performed using GraphPad Prism 6.0 software. Unpaired data (obese versus the control group) were compared using the nonparametric Mann–Whitney test. Paired data (obese patients before and after surgery) were analyzed using the nonparametric Wilcoxon test. Correlation analysis was conducted using the Spearman test for nonparametric variables. Data are expressed as mean ± SD or mean ± SEM. *p* < 0.05 was considered significant.

## 3. Results

### 3.1. Lipid Profile and Inflammation Markers

Table 1 shows the anthropometric and clinical characteristics, lipid profile, and inflammatory markers of the control subjects and the obese patients at baseline and at 6 and 12 months after bariatric surgery. Within the obese group, nine (69%) patients underwent sleeve gastrectomy, and four (31%) patients underwent gastric bypass. At baseline, obese patients presented similar levels of total cholesterol, LDLc, and apoB as the control group, but their triglycerides and VLDLc were higher, and their HDLc levels were lower than those in the control group. Bariatric surgery decreased TG and VLDLc at 6 months after surgery, and these levels remained stable, but were still higher compared with those of the control group. By contrast, the effect of surgery on HDLc levels was deferred, and the increase was only statistically significant at 12 months after surgery. Total cholesterol, LDLc, and apoB slightly decreased at 6 months but showed a rebound effect, with an increment between 6 and 12 months after surgery. No difference in NEFA concentration was observed among the groups.

The inflammatory biomarker hsCRP suggested increased systemic inflammation in the obese patients at baseline, with high hsCRP levels compared with the control group. Surgery progressively decreased these levels, albeit without reaching the same concentration as that in the control group.

### 3.2. Changes in VLDL Composition

The analysis of VLDL composition showed that the VLDL from obese patients contained more triglycerides and less apoB than the VLDL from the control group (Figure S1), which is suggestive of the larger VLDL particles in the obese group. In addition, the apoE content was decreased in the obese patients at baseline. The only effect of bariatric surgery was an increase in the apoE content in VLDL, reaching the levels of control subjects.

### 3.3. Changes in LDL Composition and Function

**Figure 1:**
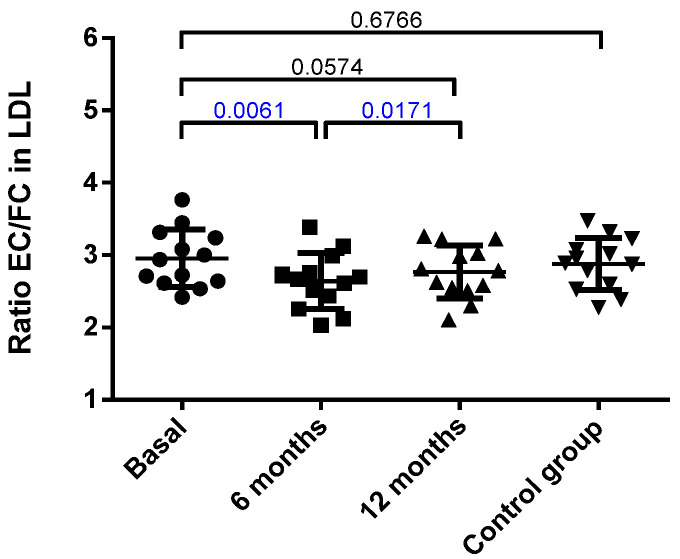
Ratio of esterified cholesterol (EC) versus free cholesterol (FC) in LDL. Data were calculated from the % of mass of each component, as described in the Methods, and are expressed as mean ± SD. Bars indicate P. Blue values indicate statistically significant differences.

The LDL composition is shown in Figure S2. No difference between the obese patients at baseline and the control group was observed, and most of the main components of LDL remained unchanged. The only significant modification was an increase in free cholesterol, which was almost significant at 6 months and reached statistical difference at 12 months. Accordingly, when the rate of cholesterol esterification was assessed by measuring the ratio of esterified cholesterol/free cholesterol (EC/FC) in LDL, a decrease in such ratio was observed at 6 months after surgery (Figure 1).

**Figure 2:**
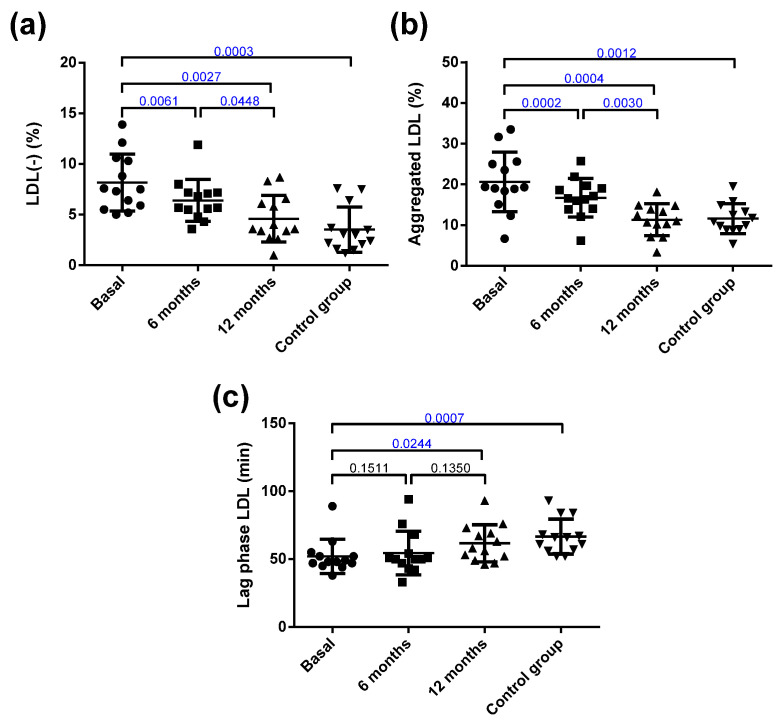
Qualitative characteristics of LDL. (**a**) Proportion of electronegative LDL (LDL(-)) determined by anion-exchange chromatography. (**b**) Susceptibility to aggregation. The proportion of aggregated LDL was determined by gel filtration chromatography after in vitro lipolysis with SMase. (**c**) Susceptibility to oxidation induced by CuSO_4_ incubation, expressed as lag phase time. Data are expressed as mean ± SD. Bars indicate P. Blue values indicate statistically significant differences.

Despite this, the only change detected after surgery in the main components of LDL was in the EC/FC ratio. The LDL from obese patients presented important alterations in their atherogenic properties compared with those from the control group (Figure 2). Thus, the LDL from the obese group was more susceptible to oxidation than the LDL from the controls. In addition, the proportion of the inflammatory plasma subfraction electronegative LDL (LDL(-)) was increased, and the susceptibility to in vitro induced aggregation was higher in the obese patients than in the controls. Interestingly, bariatric surgery reverted all these abnormalities in LDL to values similar to those shown by the control group.

**Figure 3:**
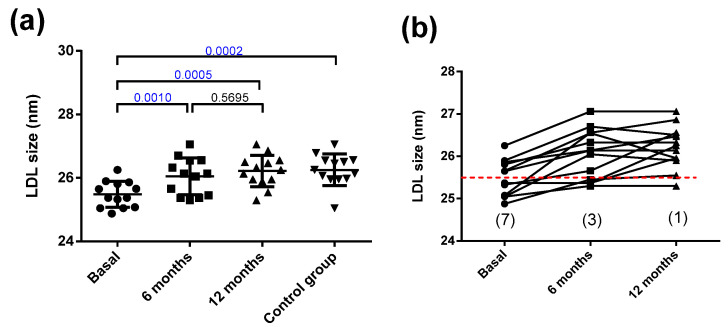
LDL size. (**a**) LDL size determined by non-denaturing gradient gel electrophoresis. (**b**) Individual changes in LDL size after bariatric surgery. The dashed red line indicates 25.5 nm, the threshold between LDL subfraction phenotypes A and B, and numbers in parentheses indicate the number of patients with the atherogenic phenotype B. Data are expressed as mean ± SD. Bars indicate P. Blue values indicate statistically significant differences.

On the other hand, the size of LDL was smaller in the obese patients than in the controls (Figure 3a). Bariatric surgery increased the LDL size to normal levels at 6 months, and this remained stable for 12 months. The small size of LDL in the obese subjects suggests a higher prevalence of subjects presenting the atherogenic LDL subfraction phenotype B than would be expected from the relatively near-normal values of plasma triglycerides (all patients were below 2.4 mmol/L). Phenotype B indicates the predominance of small LDL particles (diameter below 25.5 nm), and is considered as the atherogenic phenotype of LDL. Specifically, seven obese patients presented phenotype B. Six months after surgery, only three patients displayed phenotype B, and after one year of surgery, only one patient had phenotype B (Figure 3b). Figure S3 shows a representative GGE that includes samples from one obese patient at the three time points and one control subject.

### 3.4. Changes in HDL Composition and Function

**Figure 4:**
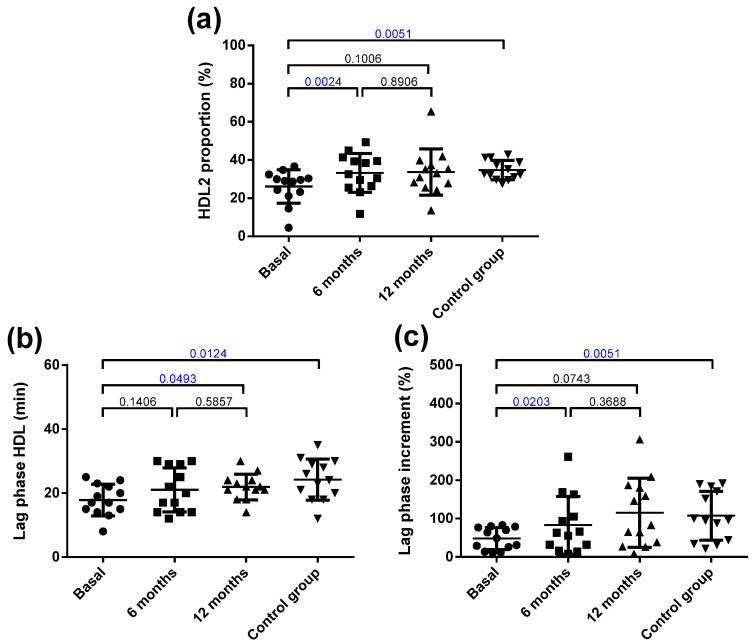
Qualitative characteristics of HDL. (**a**) Proportion of HDL2 (large HDL) determined by non-denaturing gradient gel electrophoresis. (**b**) Susceptibility to oxidation induced by CuSO_4_ incubation, expressed as lag phase time. (**c**) Antioxidant capacity of HDL to prevent the oxidation of LDL, expressed as an increment of the lag phase time. Data are expressed as mean ± SD. Bars indicate P. Blue values indicate statistically significant differences.

The HDL from the obese patients had less free and esterified cholesterol and more apoC-III compared with that from the controls (Figure S4). Bariatric surgery normalized cholesterol and apoC-III content in HDL. Surgery also induced a decrease in apoA-II content at 6 months, which redounded in a decrease in total protein. Regarding the qualitative characteristics of HDL, obese patients had a low predominance of large HDL particles (HDL2) compared with the control group (Figure 4a). Surgery normalized the relative proportion of HDL2/HDL3 at 6 months. Figure S3 shows a representative GGE that includes one obese patient at the three time points and one control subject.

Regarding oxidative properties, the HDL from the obese patients was more susceptible to oxidation than the control HDL was (Figure 4b), and its capacity to inhibit the oxidation of LDL (prolongation of the lag phase) was also decreased (Figure 4c). Bariatric surgery improved the resistance to oxidation of HDL at 12 months and its capacity to prevent the oxidation of LDL at 6 months. Figure S5 shows representative kinetics of the antioxidant capacity of the HDL from one obese patient at the three time points and one control subject.

### 3.5. Distribution of Lp-PLA_2_ Activity in Plasma

**Figure 5:**
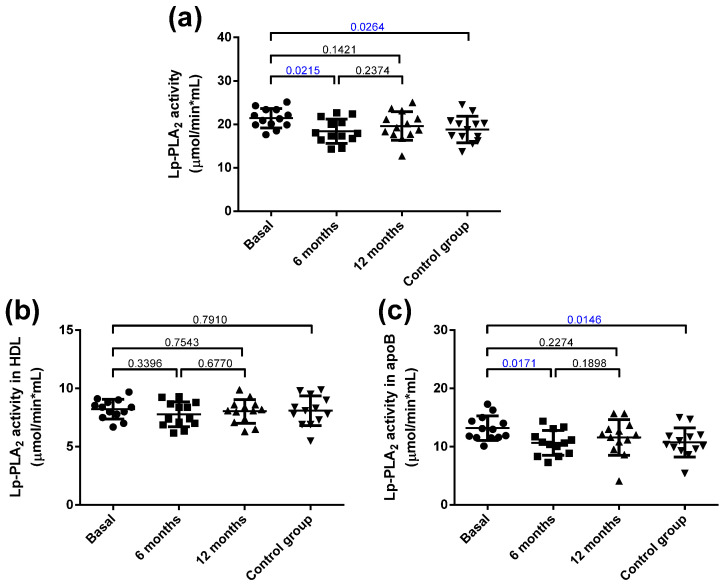
Total Lp-PLA_2_ activity and distribution in lipoproteins. (**a**) Total plasma activity. (**b**) Lp-PLA_2_ activity associated with HDL. (**c**) Lp-PLA_2_ activity associated with apoB-containing lipoproteins. Data are expressed as mean ± SD. Bars indicate P. Blue values indicate statistically significant differences.

The total Lp-PLA_2_ activity was increased in the obese group at baseline compared with the control group (Figure 5a), and it decreased 6 months after bariatric surgery. These differences were mainly attributed to increased activity in apoB-containing lipoproteins, but not in HDL (Figure 5b,c).

### 3.6. Distribution of Apolipoprotein J (apoJ) in Plasma

**Figure 6:**
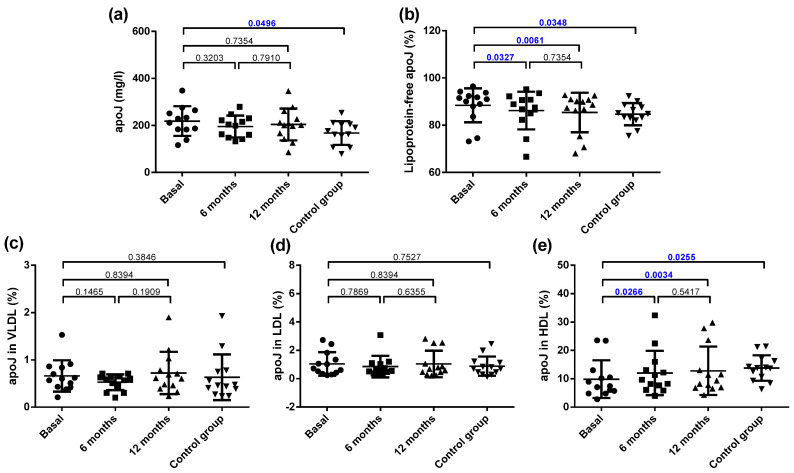
Plasma apoJ concentration and distribution in lipoproteins. (**a**) Total plasma apoJ. (**b**) Proportion of apoJ not bound to lipoproteins. (**c**) Proportion of apoJ bound to VLDL. (**d**) Proportion of apoJ bound to LDL. (**e**) Proportion of apoJ bound to HDL. Data are expressed as mean ± SD. Bars indicate P. Blue values indicate statistically significant differences.

The total plasma concentration of apoJ was increased in the obese patients compared with the controls, and no effect of bariatric surgery was observed (Figure 6a). It has been reported that most of apoJ in plasma is present in free form, not bound to lipoproteins, and only a proportion ranging from 5–30% is transported associated to lipoproteins. The proportion of apoJ not bound to lipoproteins (free form) was higher in the obese group than in the control group (Figure 6b). Since the apoJ carried by lipoproteins is bound mainly to HDL, this difference was mainly due to the decreased content of apoJ associated with HDL, whereas no difference was observed in the apoJ content in LDL and VLDL (Figure 6c–e). Bariatric surgery increased the apoJ content in lipoproteins at 6 months, mainly because of an increase in HDL (Figure 6b,e).

### 3.7. Correlation Analysis

To confirm the robustness of the observed changes and define the parameters they depend on, a correlation analysis was performed using the total number of samples analyzed. Table S1 shows the statistical correlations between lipoprotein profile, lipoprotein function, Lp-PLA_2_ activity, and apoJ-related parameters. The main findings are as follows: plasma triglycerides and VLDLc correlated negatively with LDL size and susceptibility to oxidation, whereas HDLc was positively associated with LDL size and negatively with LDL(-) proportion and LDL susceptibility to aggregation. Interestingly, the parameters of LDL function did not show associations with total cholesterol or LDLc; instead, they were mainly associated with TG and VLDLc. CRP, a marker of systemic inflammation, is strongly associated with most parameters of LDL and HDL function. This association is particularly strong with LDL(-), an inflammatory subfraction of LDL present in blood. Lp-PLA_2_ activity correlated negatively with the susceptibility to oxidation of HDL, and this activity associated to apoB also correlated negatively with the susceptibility to oxidation of HDL and with its antioxidant capacity. Finally, apoJ plasma concentration was negatively associated with the susceptibility to oxidation of LDL, pointing to a protective role of apoJ.

Associations between the lipoprotein function and its composition were also tested. Table S2 shows the correlations in LDL. The only function of LDL clearly associated with its composition was the LDL susceptibility to oxidation, which was dependent on the degree of cholesterol esterification (the higher the free cholesterol content, the higher the resistance to oxidation). Weak associations were also observed between the proportion of LDL(-) and the phospholipid content, and between LDL size and free cholesterol content.

Regarding HDL (Table S3), the susceptibility to oxidation correlated positively with the content of triglycerides and apoJ, and negatively with apoA-I. The antioxidant capacity of HDL correlated positively with cholesterol, triglycerides, and apoJ, and negatively with total protein content. The proportion of HDL2 correlated positively with cholesterol, triglycerides, apoA-I, and apoJ, and negatively with total protein content

Lipoprotein function parameters are strongly intertwined with one another (Table S4), with a higher proportion of LDL(-) being statistically associated with higher LDL susceptibility to aggregation, smaller LDL and HDL size, increased susceptibility to the oxidation of LDL and HDL, and decreased HDL antioxidant capacity. The parameters of HDL function positively correlated with the content of apoJ in HDL, supporting the role of apoJ in the maintenance of the antiatherogenic properties of HDL.

## 4. Discussion

This study reveals numerous functional alterations in lipoproteins isolated from morbidly obese subjects. The main findings are: (1) VLDL from obese patients at baseline presented only moderate alterations in composition, suggestive of a larger size, and bariatric surgery increased apoE content; (2) despite the similar LDL composition in both groups, the LDL from obese patients showed numerous abnormalities in their function, which improved after bariatric surgery; (3) the protective properties of HDL from obese patients were compromised when compared with the HDL from control subjects, but these characteristics improved after surgery; (4) the total and apoB-associated Lp-PLA_2_ activities were high in the obese patients and decreased 6 months after surgery; (5) plasma apoJ levels were increased in the obese patients compared with the control group, but the amount of apoJ transported by HDL was lower in the obese patients. Bariatric surgery normalized the cargo of apoJ in HDL.

VLDL particles from obese subjects contained more triglycerides and less apoB, indicating their larger size. The finding that bariatric surgery promoted a progressive increase in the content of apo E in VLDL, reaching the values of the control group, suggests that VLDL clearance from blood could be faster after surgery, in accordance with the role of apoE as a ligand for lipoprotein receptors [33,34]. This accelerated VLDL catabolism could add to the decreased availability of NEFA in the liver because of loss of visceral adipose tissue, as major mechanisms behind the decrease in VLDLc levels. To our knowledge, this is the first report of increased apoE content in the VLDL of patients with obesity after bariatric surgery.

LDL presented several potential atherogenic traits when isolated from obese patients, including a decreased size, increased susceptibility to oxidation and aggregation, and a high proportion of LDL(-). Bariatric surgery succeeded in reverting these alterations. Interestingly, the improvement in the qualitative characteristics of LDL occurred without changes in the plasma levels of LDLc and in the major components of LDL. Only a minor change in the free cholesterol content and the EC/FC ratio was observed after bariatric surgery. This change could underlie the improvement of LDL susceptibility to oxidation, as suggested by the correlation analysis, which showed the association of LDL susceptibility to oxidation with the content of free cholesterol, which was negative with the degree of cholesterol esterification, in accordance with previous reports [35]. Another factor strongly related to the improvement in LDL susceptibility to oxidation is the enlargement of LDL particles after bariatric surgery, as small LDL particles are highly oxidizable [35,36].

LDL(-) is a pool of all the modified forms of LDL present in blood with inflammatory and apoptotic properties [37,38]. The LDL(-) proportion was increased in adult obese patients, which agrees with previous findings of increased LDL(-) in obese adolescents [39]. In addition, we demonstrate for the first time that bariatric surgery decreased LDL(-) proportion. LDL(-) correlated with susceptibility to aggregation, which is explained by LDL(-) being highly susceptible to aggregation [40]. LDL aggregation in the subendothelial space is an early event in atherogenesis [41], and the susceptibility of LDL to aggregation has been related to an accelerated development of atherosclerosis [42]. LDL(-) was associated with all functional parameters of HDL, its being proportion higher when the size, susceptibility to oxidation, or antioxidant capacity of HDL was low. This observation emphasizes the role of HDL in the prevention of LDL modification [43].

Our results indicate that in obese patients at baseline, the HDL particles are smaller and more susceptible to oxidation, and they have partially lost their capacity to prevent LDL oxidation. Bariatric surgery, not only increased plasma HDLc levels, but also reverted these functional abnormalities. An abnormal size distribution of HDL in obesity has been reported previously, and interventions directed to weight loss, including diet and surgery, increase the size of HDL, shifting its distribution to larger particles [10,11,12,13,14,15,16,19,21]. Our results suggest that cholesterol esterification in HDL after surgery is the key element favoring the formation of large HDL particles and the improvement in their antioxidative properties. Indeed, correlation analysis showed a positive association of the antioxidant capacity of HDL with the content of esterified cholesterol and the esterified/free cholesterol ratio. We also found negative associations between the susceptibility to oxidation and the apoA-I content (the longer the lag phase, the lower the apoA-I content), and between the antioxidant capacity and the protein content in HDL (the higher the increment of the lag phase, the lower the protein content). In both cases, the explanation is related to the size of HDL, since HDL2, which contains less protein and apoA-I, is positively associated with the antioxidant capacity of HDL.

Another relevant finding is the alteration in the content of some apolipoproteins in HDL. The HDL from the obese patients contained more apoC-III and less apoJ than the HDL from the controls, and bariatric surgery normalized the cargo of both apolipoproteins in HDL. This finding is relevant because similar alterations in apoC-III and apoJ content were observed in HDL from coronary patients by Riwanto et al. [44].

Regarding apoJ, our results demonstrate that compared with the control group, the obese subjects presented increased plasma concentration of apoJ, but a decreased proportion bound to HDL. Bariatric surgery reverted both alterations. These findings agree with previous reports on the associations between apoJ, insulin resistance [45], and adiposity [46], and indicate an altered distribution of apoJ among lipoproteins in obesity [47,48]. ApoJ prevents the aggregation of LDL [49], inhibits the apoptosis induced by modified LDL [50], and stabilizes several proteins in HDL with antioxidant properties, including apoA-I and paraoxonase 1 [51]. This concurs with our observation that the content of apoJ in HDL positively correlates with the protective properties of HDL, including size, susceptibility to oxidation, and antioxidant capacity. Therefore, apoJ appears to be a key factor contributing to the preservation of the antiatherogenic properties of HDL.

**Figure 7:**
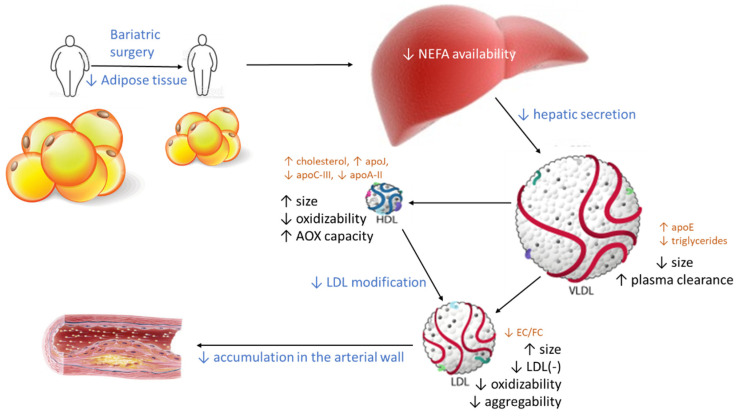
Effect of bariatric surgery on the qualitative properties of lipoproteins. The loss of visceral adipose tissue after surgery decreases the availability of NEFA in the liver and promotes lower VLDL secretion. This VLDL contains more apoE, which favors VLDL clearance in plasma, and less triglycerides, indicative of smaller VLDL particles. Therefore, the catabolism of smaller VLDL particles produces larger LDL and HDL particles with altered contents of some major components. Both large LDL and HDL are more resistant to oxidative modifications, and HDL has an increased capacity to prevent further modifications in LDL, both in plasma and in the arterial wall. Consequently, the deposition of LDL in arteries should decline after bariatric surgery. Blue text: events occurring after bariatric surgery. Black text: changes in the properties of lipoproteins. Brown text: changes in composition. AOX: antioxidant.

From these findings, a scheme can be drawn (Figure 7) in which the decrease in central adiposity after bariatric surgery shifts the altered qualitative characteristics of LDL and HDL to a non-atherogenic phenotype. The driving force could be a decrease in the available free fatty acids in the liver because of the loss of adipose tissue. This decreases hepatic VLDL secretion, which adds to the increased apoE content in VLDL, favoring its receptor-mediated clearance. Consequently, VLDL particles are smaller, and their metabolism in blood generates large LDL particles, which are less susceptible to oxidation. In parallel, the size and composition of HDL particles becomes closer to the composition of healthy subjects. As a result, the capacity of HDL to prevent modification improves, and less modified LDL particles are found in circulation. As a whole, the functions of LDL and HDL in obese patients are normalized after bariatric surgery. Of note, the improvement of the lipoprotein function after surgery occurs in some cases without a complete normalization of the lipoprotein composition and size, suggesting a threshold beyond which lipoprotein function becomes normal.

The main limitation of our study is the small sample size in each group. Nevertheless, as a whole, the alterations observed in the composition and function of each lipoprotein fraction are coherent and metabolically connected, and they support the consistency of the achieved results. Another limitation is that a group of obese subjects displaying dyslipidemia were not compared with the obese subjects included in the present study, which would have made it possible to estimate the specific effect of hyperlipemia on alterations in lipoprotein function. Finally, no measures of atherosclerosis, either clinical or subclinical were assessed, therefore, the impact of the findings affecting the function of lipoproteins on developing arteriosclerosis requires further studies, analyzing this particular aspect.

## 5. Conclusions

In summary, our study shows multiple qualitative modifications in the lipoproteins from obese subjects, which are reverted, in part, by the weight loss induced after bariatric surgery. A decreased hepatic secretion of VLDL after surgery elicits a cascade of metabolic changes in LDL and HDL that improve their functional properties. Our study also reveals the intertwined relationship among the main components of the three main lipoprotein fractions in obese patients. Further studies are warranted (1) to confirm our findings in larger cohorts of patients with severe obesity, and (2) to relate the alterations of lipoprotein function with the development of atherosclerosis or the emergence of clinical events.

## Figures and Tables

**Table 1:** Anthropometric and clinical characteristics, lipid profile, and inflammatory markers.

Parameters	Obese (*n* = 13)				Control Group (*n* = 13)
Age (years)	51.3 ± 8.1			42.5 ± 11.6	
Gender (M/F)	8/5			6/7	
	Baseline	6 months	12 months		
BMI (kg/m^2^)	42.7 ± 3.9	34.1 ± 3.0 ^a^	30.0 ± 3.6 ^a,b^		22.6 ± 2.1 ^a,b,c^
% TWL		20.02 ± 6.42	28.87 ± 8.99 ^b^		
Fasting glucose (mmol/L)	6.68 ± 2.04	5.61 ± 1.30 ^a^	5.32 ± 0.97 ^a^		4.74 ± 0.47 ^a,b^
HbA1c (%)	5.91 ± 0.80	5.58 ± 0.59	5.51 ± 0.73 ^a^		5.13 ± 0.27 ^a,b^
Cholesterol (mmol/L)	5.05 ± 0.85	4.34 ± 0.80 ^a^	5.08 ± 1.00 ^b^		5.22 ± 0.78 ^b^
Triglycerides (mmol/L)	1.45 ± 0.51	1.12 ± 0.35 ^a^	1.13 ± 0.36 ^a^		0.86 ± 0.28 ^a,b,c^
VLDL-c (mmol/L)	0.67 ± 0.24	0.51 ± 0.16 ^a^	0.51 ± 0.17 ^a^		0.40 ± 0.13 ^a^
LDL-c (mmol/L)	3.23 ± 0.76	2.67 ± 0.70	3.33 ± 0.83 ^b^		3.28 ± 0.68 ^b^
HDL-c (mmol/L)	1.16 ± 0.19	1.15 ± 0.19	1.38 ± 0.22 ^a,b^		1.54 ± 0.29 ^a,b^
ApoB (g/L)	1.06 ± 0.23	0.86 ± 0.17 ^a^	1.00 ± 0.16 ^b^		0.92 ± 0.15 ^a,b^
NEFA (mmol/L)	0.56 ± 0.28	0.66 ± 0.23	0.62 ± 0.20		0.49 ± 0.18
hsCRP (mg/L)	9.96 ± 6.71	5.28 ± 4.83 ^a^	2.88 ± 2.21 ^a^		0.84 ± 0.15 ^a,b,c^

^a^*p* < 0.05 vs. baseline. ^b^ *p* < 0.05 vs. 6 months. ^c^ *p* < 0.05 vs. 12 months. BMI: body mass index; TWL: total weight loss.

## Data Availability

The data presented in this study are available on reasonable request from the corresponding author.

## Supplementary Materials

The following are available online at https://www.mdpi.com/article/10.3390/jcm10081716/s1. Figure S1: VLDL composition. Figure S2: LDL composition. Figure S3: Representative gradient gel electrophoresis (GGE). Figure S4: HDL composition. Figure S5: Susceptibility to oxidation of LDL and HDL, and antioxidant capacity of HDL. Table S1: Correlation analysis between lipid profile and total plasma parameters, and the lipoprotein function variables. Table S2: Correlation analysis between LDL composition, and LDL function. Table S3: Correlation analysis between HDL composition, and HDL function. Table S4: Correlation analysis between the parameters of lipoprotein function.
