# Supplementary material for: Changes in the Composition and Function of Lipoproteins after Bariatric Surgery in Patients with Severe Obesity
Source: J Clin Med. 2021 Apr 16;10(8):1716. doi: 10.3390/jcm10081716 (PMC8071565; doi:10.3390/jcm10081716)
Supplement: Supplementary file 1 [file jcm-10-01716-s001.zip › jcm-1197561-supplementary.pdf]

Changes in the composition and function of lipoproteins after bariatric surgery in patients with severe obesity

Idoia Genua[1,2,a], Núria Puig[2,3,4,a], Inka Miñambres[1,a], Sonia Benítez[3], Pedro Gil[1], Margarida Grau-Agramunt[3], Andrea Rivas-Urbina[2,3], Carme Balagué[2,4], Sonia Fernández-Alanin[4], Álvaro Garcia-Osuna[3], Antonio Pérez[1,4*], José Luis Sánchez-Quesada[3,4*]

Supplementary Material.

Supplementary Figure S1. VLDL composition. Data are calculated from the % of mass of each component, as described in Methods, and are expressed as mean ± SD. Bars indicate P. Blue values indicate statistically significant differences.

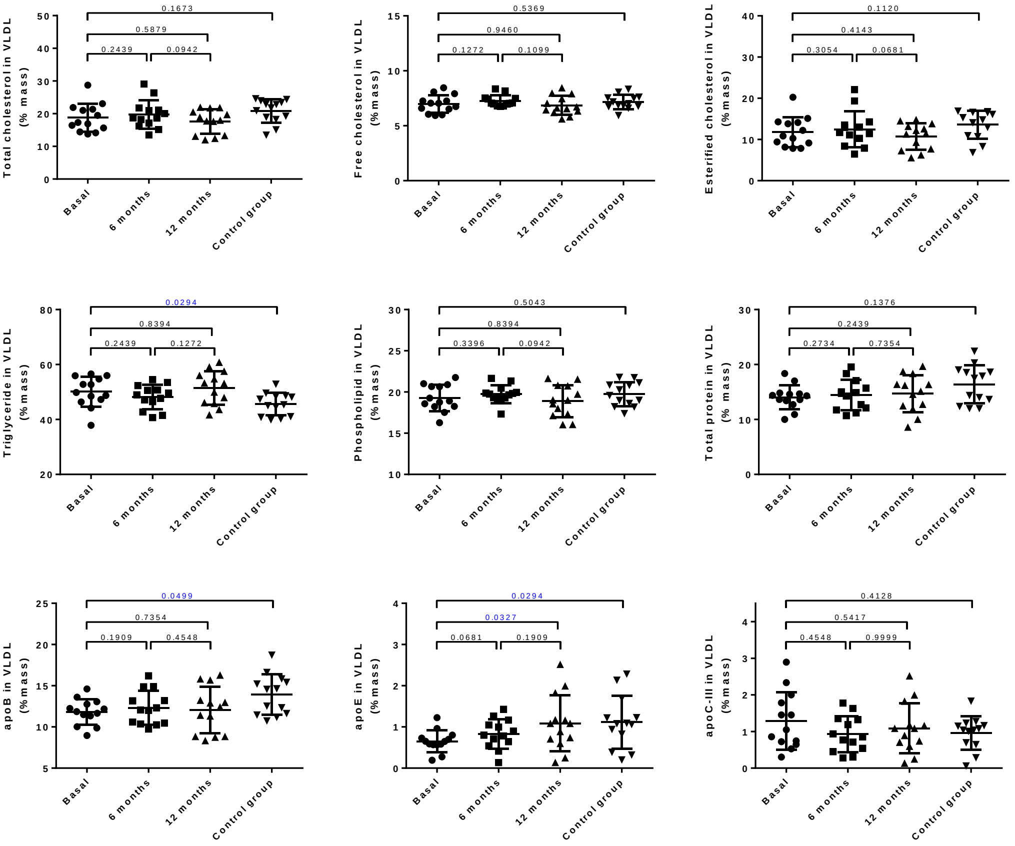

Supplementary Figure S2. LDL composition. Data are calculated from the % of mass of each component, as described in Methods, and are expressed as mean ± SD. Bars indicate P. Blue values indicate statistically significant differences.

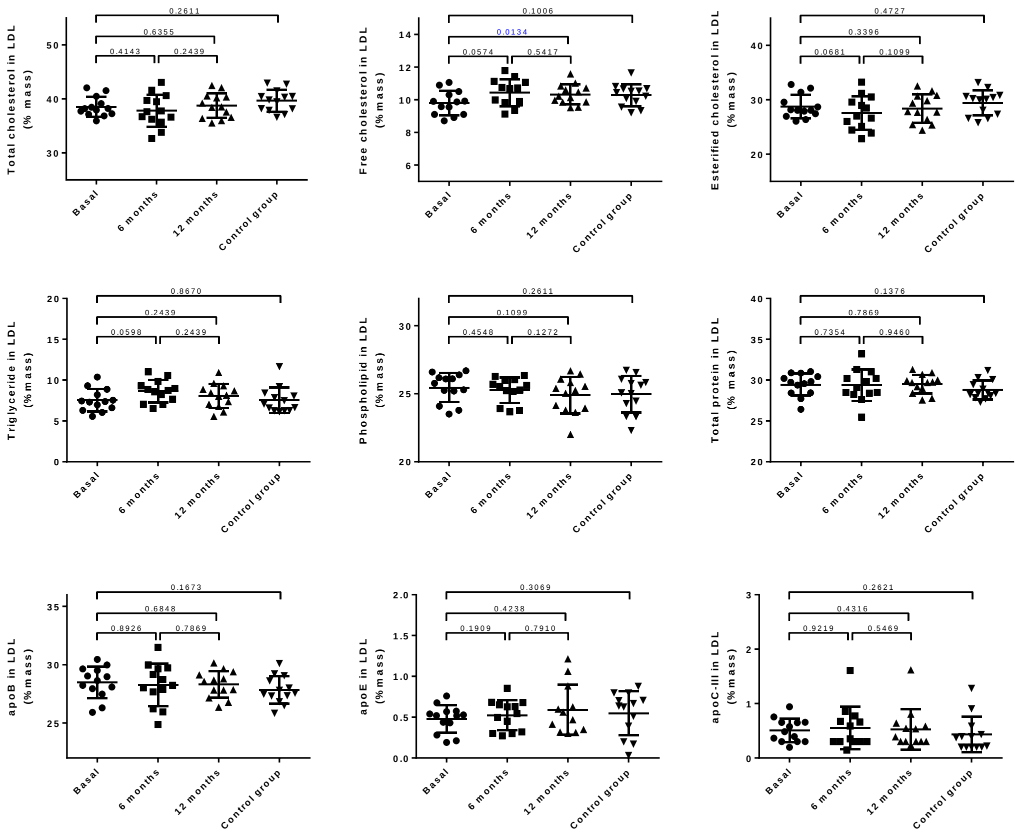

Supplementary Figure S3. Representative gradient gel electrophoresis (GGE). Electrophoresis was essentially performed as described in reference 26, using an acrylamide gradient of 2.5-16%. Briefly, plasma was pre-stained with Sudan black for lipid staining, and electrophoresis was conducted at 100 V for 6 hours. LDL and HDL bands were scanned by densitometry.

LDL size was determined using a pool of plasma containing four LDL bands of known size (24.5, 25.1, 26.3, and 28.1 nm) as a standard. The diameter of standard LDL bands was previously assessed by electron microscopy. The dashed red line indicates 25.5 nm, the threshold between phenotype A (>25.5) and phenotype B (<25.5).

The relative distribution of HDL subfractions was calculated from the densitometry analysis of the bands corresponding to HDL2 and HDL3.

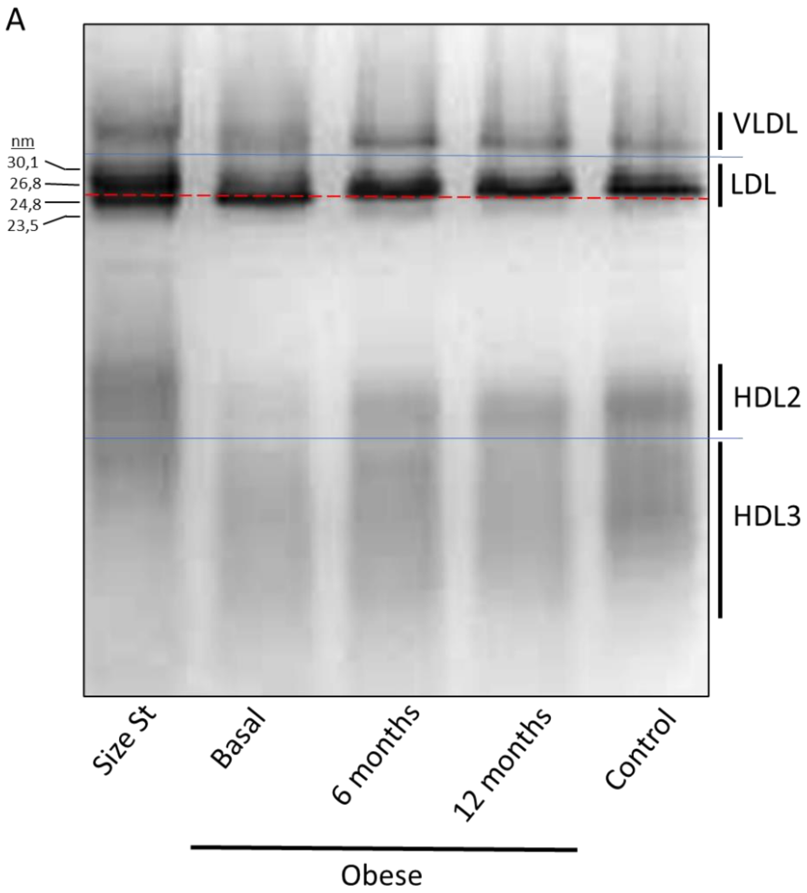

Supplementary Figure S4. HDL composition. Data are calculated from the % of mass of each component, as described in Methods, and are expressed as mean ± SD. Bars indicate P. Blue values indicate statistically significant differences.

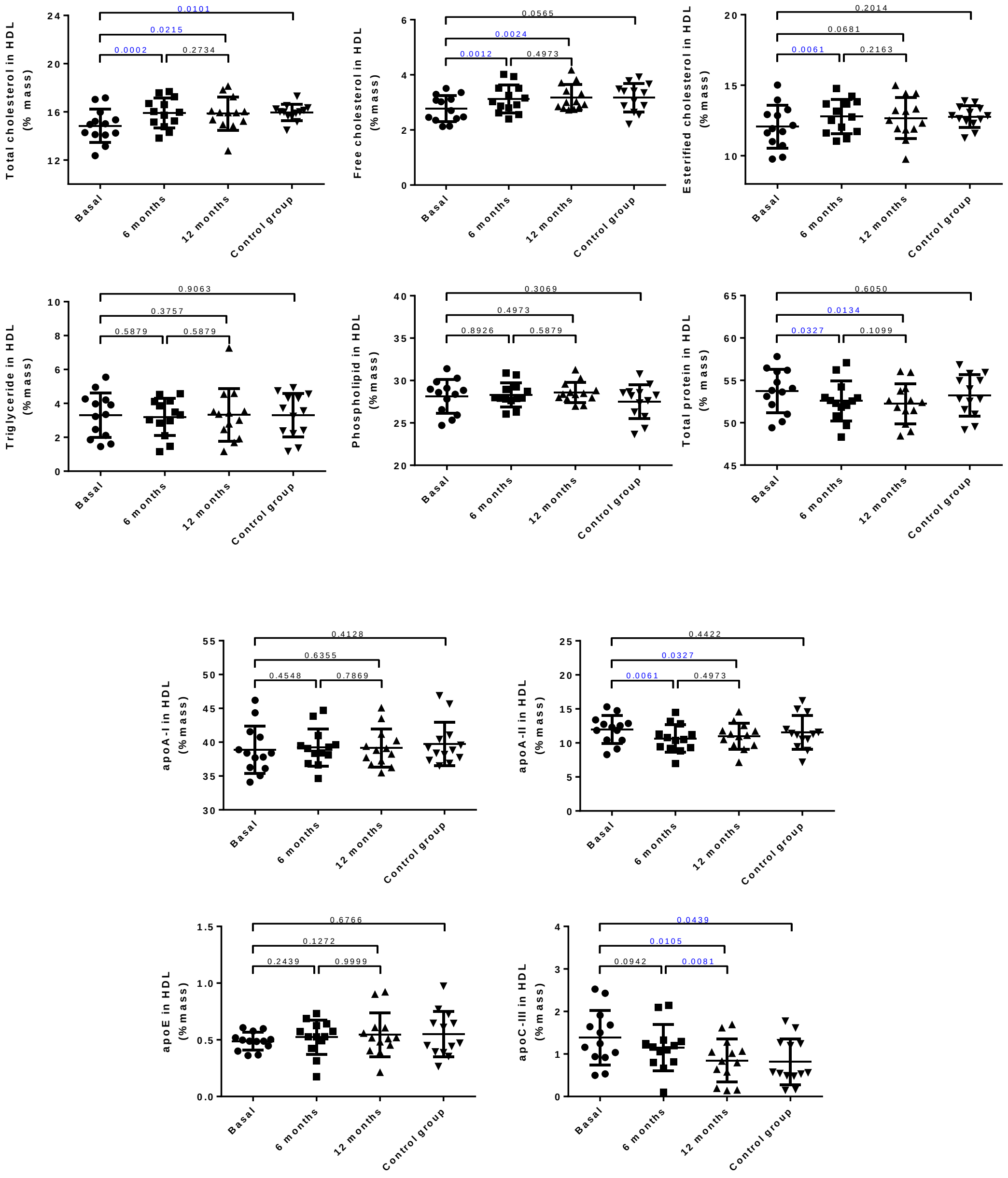

Supplementary Figure S5. Susceptibility to oxidation of LDL and HDL, and antioxidant capacity of HDL.

A) Characteristic sigmoidal curves of LDL, HDL, and HDL+LDL obtained after the oxidation process induced by $CuSO_4$ (5 µM) addition. The lag phase time of lipoproteins is calculated from the intersection point between the maximal slope of the curve and initial absorbance.

B) The kinetics of HDL alone is subtracted from the kinetics of HDL+LDL to obtain the curve (HDL+LDL)-HDL that allows to assess the increment of lag phase time induced by the presence of HDL.

C) Representative kinetics of the (LDL+HDL)-HDL curves of one obese patient at the 3 time points and one control subject. Results are expressed as the increment of lag phase time versus the lag time of LDL alone.

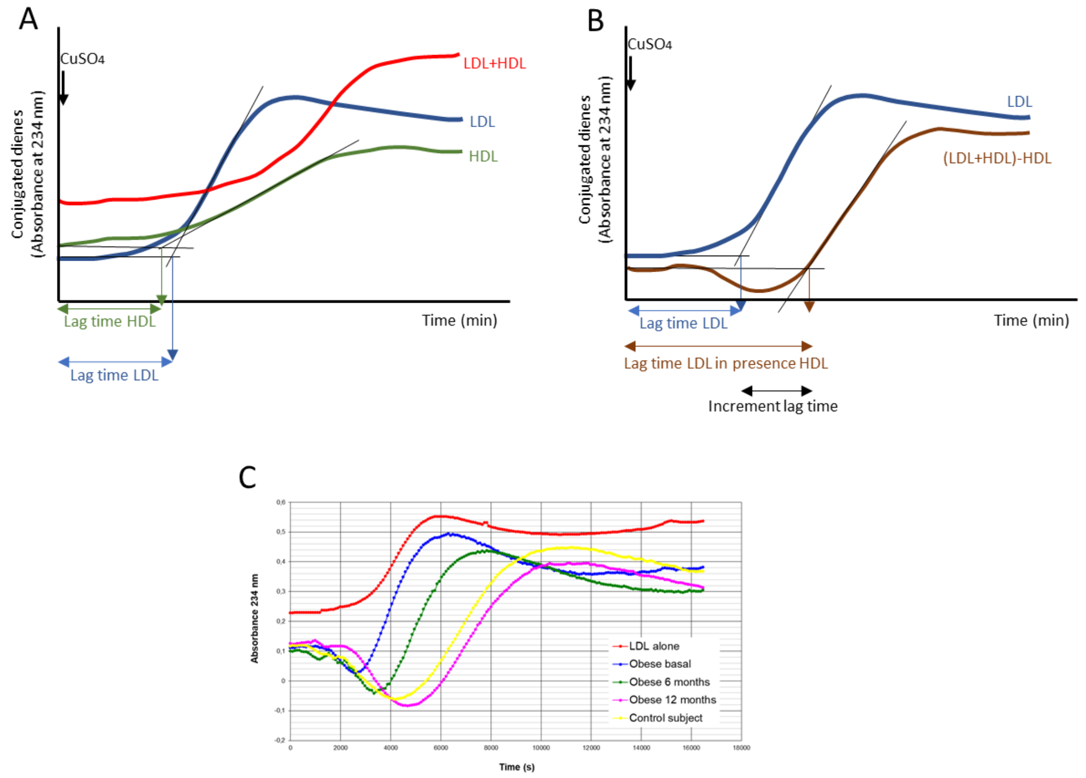

Supplementary Table S1. Correlation analysis between lipid profile and total plasma parameters, and the lipoprotein function variables.

	LDL(-)<br>(%)	Susc ag LDL<br>(% agLDL)	LDL size<br>(nm)	Susc ox LDL<br>(min)	Susc ox HDL<br>(min)	AOX HDL<br>(Δ min)	HDL2<br>(%)
TC<br>(mmol/L)	0,080909<br>-0,244304	0,140294<br>-0,207314	0,769159<br>-0,041693	0,662000<br>-0,062072	0,355430<br>-0,130778	0,491647<br>0,097508	0,144449<br>0,205213
TG<br>(mmol/L)	0,285858<br>0,150818	0,607059<br>0,072996	**0,001179**<br>**-0,437572**	**0,034166**<br>**-0,294341**	0,321669<br>-0,140159	0,934982<br>-0,011594	0,975554<br>-0,004355
VLDLc<br>(mmol/L)	0,282792<br>0,1517708	0,630640<br>0,068259	**0,001079**<br>**-0,440682**	**0,032145**<br>**-0,297591**	0,309623<br>-0,1436552	0,968112<br>-0,0056818	0,970273<br>0,005296
LDLc<br>(mmol/L)	0,109401<br>-0,224616	0,200813<br>-0,180323	0,534975<br>-0,0880103	0,290978<br>-0,149243	0,095824<br>-0,233427	0,609765<br>0,072449	0,493010<br>0,097203
HDLc<br>(mmol/L)	**0,001407**<br>**-0,431411**	**0,017546**<br>**-0,328159**	**0,048427**<br>**0,280512**	0,069941<br>0,253360	0,141136<br>0,2068843	0,537733<br>0,087418	0,306918<br>0,144452
ApoB<br>(g/L)	0,974108<br>0,004612	0,364420<br>-0,128373	0,128047<br>-0,213791	0,111487<br>-0,223338	0,101052<br>-0,229926	0,906579<br>0,016678	0,952799<br>-0,008412
CRP<br>(mg/L)	**0.000096**<br>**0,523347**	**0,004954**<br>**0,391308**	**0,009144**<br>**-0,365046**	**0,006382**<br>**-0,380708**	**0,000153**<br>**-0,510350**	**0,000956**<br>**-0,452976**	**0,000227**<br>**-0,498750**
Lp-PLA_2<br>(µmol/min/mL)	0,754422<br>-0,044438	0,286462<br>0,150632	0,387645<br>-0,151000	0,285272<br>-0,122323	**0,022716**<br>**-0,315478**	0,065917<br>-0,256966	0,516657<br>-0,091976
ApoB-Lp-PLA_2<br>(µmol/min/mL)	0,837365<br>-0,029168	0,176276<br>0,190439	0,247191<br>-0,163364	0,535634<br>-0,087868	**0,033060**<br>**-0,296098**	**0,048023**<br>**-0,275547**	0,511419<br>-0,093123
ApoJ<br>(mg/L)	0,241487<br>0,170498	0,207086<br>-0,183432	0,514967<br>-0,095265	**0,003735**<br>**-0,406670**	0,114897<br>-0,228131	0,528583<br>0,092213	0,838129<br>-0,029950

Correlation analysis was conducted using the Spearman test for nonparametric variables. $P < 0.05$ was considered significant. Upper number in each cell indicates ***P*** significance. Lower number in each cell indicates ***r*** correlation coefficient. Bold numbers indicate significant correlations.

LDL(-): electronegative LDL; Susc ag LDL: susceptibility to aggregation of LDL; Susc ox LDL: susceptibility to oxidation of LDL; Susc ox HDL: susceptibility to oxidation of HDL; AOX HDL: antioxidant capacity of HDL; TC: Total cholesterol; TG: triglycerides; VLDLc: VLDL cholesterol; LDLc: LDL cholesterol; HDLc: HDL cholesterol; Apo: apolipoprotein; CRP: C-reactive protein; Lp-PLA_2: lipoprotein-associated phospholipase A_2; apoB-Lp-PLA_2: apoB-associated phospholipase A_2.

Supplementary Table S2. Correlation analysis between LDL composition, and LDL function.

	LDL(-) (%)	Susc ag LDL (% agLDL)	LDL size (nm)	Susc ox LDL (min)
Total cholesterol (%)	0,140160<br>-0,207382	0,293481<br>-0,148480	0,744571<br>0,046282	0,191436<br>-0,184079
Triglycerides (%)	0,594803<br>-0,075489	0,696405<br>0,055413	0,386538<br>0,122606	0,386099<br>-0,122719
Phospholipids (%)	**0,042289**<br>**0,282701**	0,160600<br>0,197447	0,766083<br>-0,042264	0,108620<br>0,225099
Free cholesterol (%)	0,541407<br>-0,086633	0,320491<br>0,140496	**0,049034**<br>**0,274359**	**0,002202**<br>**0,415288**
Esterified cholesterol (%)	0,235984<br>-0,167246	0,297004<br>-0,147412	0,835375<br>-0,029529	0,036620<br>-0,290603
Ratio E/F cholesterol	0,547416<br>-0,085354	0,208927<br>-0,177172	0,161912<br>-0,196841	**0,001870**<br>**-0,421258**
ApoB (%)	0,076819<br>0,247561	0,628055<br>0,068775	0,408234<br>-0,1171368	0,125057<br>0,215442
ApoE (%)	0,683561<br>0,058477	0,265364<br>-0,158909	0,175245<br>0,192806	0,105866<br>0,229089
ApoC-III (%)	0,084481<br>0,241564	0,087188<br>0,239548	0,676957<br>-0,059162	0,026814<br>0,307060

Correlation analysis was conducted using the Spearman test for nonparametric variables. $P < 0.05$ was considered significant. Upper number in each cell indicates ***P*** significance. Lower number in each cell indicates ***r*** correlation coefficient. Bold numbers indicate significant correlations.

LDL(-): electronegative LDL; Susc ag LDL: susceptibility to aggregation of LDL; Susc ox LDL: susceptibility to oxidation of LDL; Ratio E/F: ratio esterified/free cholesterol; Apo: apolipoprotein.

Supplementary Table S3. Correlation analysis between HDL composition, and HDL function.

	Susc ox HDL (min)	AOX HDL (Δ min)	HDL2 (%)
Total cholesterol (%)	0,059067<br>0,263533	**0.000023**<br>**0,551331**	**0.0000008**<br>**0,623180**
Triglycerides (%)	**0,010387**<br>**0,352461**	**0.000080**<br>**0,519223**	**0,031992**<br>**0,297844**
Phospholipids (%)	0,793848<br>-0,037128	0,117937<br>-0,219499	0,107999<br>-0,225485
Free cholesterol (%)	0,118492<br>0,219176	0,061551<br>-0,261084	0,670332<br>0,060448
Esterified cholesterol (%)	0,215706<br>0,174605	**0.0000001**<br>**0,655167**	**0.000015**<br>**0,560298**
Ratio E/F cholesterol	0,419687<br>-0,114317	**0,000552**<br>**0,462704**	0,172937<br>0,191893
Total protein (%)	0,166788<br>-0,194623	**0,004643**<br>**-0,386567**	**0,023723**<br>**-0,313297**
ApoA-I (%)	**0,027549**<br>**-0,305666**	0,612273<br>-0,071942	**0,027124**<br>**0,306467**
ApoA-II (%)	0,075128<br>0,248947	0,180900<br>0,188459	0,376629<br>-0,125165
ApoE (%)	0,066361<br>0,256561	0,243361<br>0,164677	0,907934<br>0,0164354
ApoC-III (%)	0,168793<br>0,193725	0,148362<br>0,203274	0,287368<br>-0,150352
ApoJ-HDL (%)	**0,017204**<br>**0,329106**	**0.000019**<br>**0,555387**	**0,035831**<br>**0,291782**

Correlation analysis was conducted using the Spearman test for nonparametric variables. $P < 0.05$ was considered significant. Upper number in each cell indicates ***P*** significance. Lower number in each cell indicates ***r*** correlation coefficient. Bold numbers indicate significant correlations.

Susc ox HDL: susceptibility to oxidation of HDL; AOX HDL: antioxidant capacity of HDL; Ratio E/F: ratio esterified/free cholesterol; Apo: apolipoprotein; apoJ-HDL: proportion of apoJ bound to HDL.

Supplementary Table S4. Correlation analysis between the parameters of lipoprotein function.

	LDL(-) (%)	Susc ag LDL (% agLDL)	LDL size (nm)	Susc ox LDL (min)	Susc ox HDL (min)	AOX HDL (Δ min)	HDL2 (%)
LDL(-) (%)		**0.000080** **0,519226**	**0,034631** **-0,293616**	0,074779 -0,249236	**0,039169** **-0,286934**	**0,004889** **-0,384480**	**0,008877** **-0,359401**
Susc ag LDL (% agLDL)			**0,018239** **-0,326288**	0,227084 -0,170420	0,310155 -0,143499	**0,017660** **-0,327846**	0,061392 -0,261239
LDL size (nm)				**0,009327** **0,357231**	0,233997 0,167947	0,155791 0,199696	0,095665 0,233535
Susc ox LDL (min)					**0,020301** **0,321059**	0,274267 -0,154454	0,774043 -0,040787
Susc ox HDL (min)						**0,023483** **0,313810**	0,182234 0,187894
AOX HDL (Δ min)							**0,000402** **0,472662**
HDL2 (%)							

Correlation analysis was conducted using the Spearman test for nonparametric variables. $P < 0.05$ was considered significant. Upper number in each cell indicates ***P*** significance. Lower number in each cell indicates ***r*** correlation coefficient. Bold numbers indicate significant correlations.

LDL(-): electronegative LDL; Susc ag LDL: susceptibility to aggregation of LDL; Susc ox LDL: susceptibility to oxidation of LDL; Susc ox HDL: susceptibility to oxidation of HDL; AOX HDL: antioxidant capacity of HDL.
